# Survival Patterns and Mortality Causes in Patients with Invasive Ependymoma: A Retrospective Cohort Analysis from 2000 to 2019

**DOI:** 10.3390/medsci13030139

**Published:** 2025-08-16

**Authors:** Anas Elgenidy, Khaled Saad, Amir Aboelgheet, Eman F. Gad, Usama El-Shokhaiby, Thamer A. M. Alruwaili, Abdelrahman N. Abdelal, Kawashty R. Mohamed, Mohammad Bazzazeh, Mohamed Hesn, Abdelrahman H. Elshimy, Aya Sayed, Sara Magdy, Doaa Ali Gamal, Amira A. Elhoufey, Shymaa Adel Ismael, Ahmed M. Afifi, Mohamed Fahmy M. Ibrahim, Amany Ragab

**Affiliations:** 1 Department of Neurology, Faculty of Medicine, Cairo University, Cairo 12613, Egypt; 2 Pediatric Department, Faculty of Medicine, Assiut University, Assiut 71516, Egypt; 3 Department of Neurosurgery, Al Azhar University, Cairo 11884, Egypt; 4 Department of Pediatrics, College of Medicine, Jouf University, Sakaka 75741, Saudi Arabia; 5 Department of Neurology, Al-Azhar University, Assiut 71111, Egypt; 6 Faculty of Medicine, Alexandria University, Alexandria 21526, Egypt; 7 Faculty of Medicine, Al Azhar University, Damietta 25242, Egypt; 8 Medical Research Institute, Alexandria University, Alexandria 21526, Egypt; 9 Faculty of Medicine, Ain Shams University, Cairo 11591, Egypt; 10 Department of Clinical Oncology, Faculty of Medicine, Assiut University, Assiut 71516, Egypt; 11 Department of Community Health Nursing, Alddrab University College, Jazan University, Jazan 45142, Saudi Arabia; 12 Department of Community Health Nursing, Faculty of Nursing, Assiut University, Assiut 71516, Egypt; 13 Department of Neurosurgery, Al-Azhar University for Girls, Cairo 11754, Egypt; 14 Department of Surgery, University of Toledo Medical Center, Toledo, OH 44192, USA; 15 Department of Pediatrics, Al-Azhar University, Assiut 71111, Egypt

**Keywords:** ependymoma, survival analysis, mortality, central nervous system neoplasms, cause of death

## Abstract

Background: Ependymomas are primary CNS neoplasms that arise from the ependymal cells of the brain and spinal cord, accounting for 3–6% of all CNS tumors. Aims: This study provides a comprehensive analysis of ependymoma survival patterns and examines non-cancer causes of death in the US. Methods: This retrospective study used data from SEER 17 registries between 2000 and 2019 to evaluate the incidence of ependymoma, as well as the survival and mortality trends in the US. Results: A total of 3821 patients were included, with 842 (22%) deaths. The highest mortality was observed in younger patients (<18 years) within one year of diagnosis (SMR, 54.77; 95% CI, 38.95–74.88). Brain and other nervous system cancers were the leading causes of death, followed by non-cancer causes, particularly cerebrovascular diseases, pneumonia, influenza, and septicemia. The survival rates observed at one, three, and five years were 94% (95% CI: 0.94–0.95), 88% (95% CI: 0.87–0.89), and 84% (95% CI: 0.82–0.85), respectively. Conditional survival improved over time, with a three-year conditional relative survival rate of 92% after one year of diagnosis and 96% for those who survived five years. Conclusion: The death rate was highest among pediatric patients under 18 years of age. Cerebrovascular disorders were the leading non-cancer cause of death across all time intervals. The probability of surviving for three years increases for patients who have already survived one, three, or five years post-diagnosis.

## 1. Introduction

Ependymomas are neuroepithelial tumors that arise from ependymal cells that form the walls of the ventricles and the central canal of the spinal cord. While the radial glial cells in the embryonic phase are considered the primary origin of the ependymomas, their etiology has not been completely elucidated [1]. Ependymomas are a diverse group of cancers with unknown oncogenic determinants. In contrast to other central nervous system (CNS) tumors, point mutations are quite rare but significantly negatively impact the patient’s condition. However, dealing with fusion genes and copy number aberrations is critical in the generation of cancers [2]. Ependymomas constitute 3–6% of all CNS tumors, with over 50% coming from the spinal cord [3,4]. Primary spinal cord tumors are relatively infrequent, contributing to 4–10% of all CNS tumors, but ependymomas are the most common primary intramedullary spinal tumors that are seen in 30–45% of cases [3,5]. More than half of ependymomas occur in children under the age of five years [6]

Traditionally, ependymomas have been classified histologically into three grades by the World Health Organization (WHO): grade I (subependymoma and myxopapillary ependymoma), grade II (ependymoma, not otherwise specified [NOS]), and grade III (anaplastic ependymoma). However, this histological grading system has shown limited prognostic consistency. More recently, molecular classification based on DNA methylation profiling has identified nine distinct ependymoma groups across anatomical compartments (supratentorial, posterior fossa, spinal), with superior clinical and prognostic relevance. Notably, supratentorial ependymomas are now divided into ZFTA-rearranged (formerly RELA-fused) and YAP1-rearranged subtypes, while posterior fossa ependymomas are classified as PFA (posterior fossa group A) and PFB (posterior fossa group B), with PFA associated with significantly worse outcomes. Spinal ependymomas often harbor MYCN amplification or NF2 mutations. This evolving molecular framework is expected to be integrated into future WHO classifications and is increasingly guiding risk stratification and therapeutic decisions [7,8]. Thus, an integrated model was constructed based on the methylation data and clinicopathological characteristics to estimate progression-free survival. The groups with the worst 10-year overall survival were the patients diagnosed with ependymoma (PFA), ependymoma (ZFTA), and ependymoma (MYCN) tumors, with respective rates of 56%, 62%, and 32% [8]. On average, patients with ependymomas have a 5-year survival rate of 60–70% [7].

The incidence rate decreases with the patient’s age at diagnosis. The data indicate a slight male majority in ependymomas, with a ratio of 1.35:1. As the Central Brain Tumor Registry of the United States recommends, the rate of ependymomas per the human population annually is about 0.29 and 0.6 per 100,000 people [9]. Furthermore, few studies have examined the updated population-level survival of all patients diagnosed with at least one of the various subcategories of ependymoma [10]. More research that investigated ependymoma survival—depending on characteristics such as histology, tumor location, or specific age—has found contrastive survival patterns [11,12]. Also, the assessment of prognostic variables for ependymoma has remained uncertain. Earlier studies were commonly based on a cohort statistical experiment of a small number of people, and the predicted outcomes are rather diverse. Predicting patient mortality and survival times is difficult for experienced physicians, who may meet many challenges. Additionally, medical technologies differ from one institution to another, making it hard to develop a prognosis prediction of ependymoma [13]. Therefore, this study aims to provide an up-to-date and comprehensive investigation of ependymoma incidence rates, survival patterns, and mortality causes in the US.

## 2. Materials and Methods

### 2.1. Study Design and Data Source

This retrospective study utilized SEER*Stat software (version 8.4.3) to extract data from the SEER 17 registries [14,15], which cover approximately 26.5% of the US population from 1 January 2000 to 31 December 2019.

### 2.2. Study Population

The authors selected patients of known age with invasive ependymoma from 1 January 2000 to 31 December 2019; for site and morphology, we used the AYA site recode 2020 revision = 3.1.3.2 ependymoma-invasive. To eliminate any potential confounding effects from secondary malignancies and keep the emphasis on the original cancer diagnosis, this study was limited to cases that were classified as “first primary only (Sequence Number 0 or 1).” This option includes the first tumor of the patient if that tumor meets the inclusion criteria and has a sequence of 0 or 1. A sequence number equal to 0 means that it is the only tumor in the patient’s history. A sequence number equal to 1 is the first tumor of two or more tumors [16]. We also included only patients with invasive malignant behavior and excluded patients with unknown survival time or other criteria.

### 2.3. Outcomes

The authors evaluated non-cancer causes of death in individuals with invasive ependymoma, considering the following variables: sex, age at diagnosis, and race. The authors categorized causes of death by latency period: within one year, one to five years, and more than five years following the diagnosis of invasive ependymoma. Using the International Classification of Diseases 10th Revision (ICD-10)-World Health Organization (WHO) codes, the authors identified the causes of death [Supplementary Table S1]. Pregnancy, puerperium, childhood difficulties, homicide, stomach and duodenal ulcers, and perinatal disorders are included in the definition of other causes of death.

### 2.4. Statistical Analysis

We used SEER*Stat software, version 8.4.3, to calculate our survival and mortality outcomes. To estimate expected survival, we used the Ederer II method, in which the matched individuals are considered at risk until the corresponding patient dies or is censored [17]. Regarding survival outcomes, we calculated the following:

### 2.5. Relative Survival

It is the ratio of the actual survival rate of a group of patients over a certain period and the expected survival rate within the general population. Relative survival helps to isolate the effect of cancer alone on survival [17].

### 2.6. Observed Survival

It is the probability of surviving from all causes of death for a group of cancer patients. Observed survival gives a direct indication of survival rates but includes deaths from all causes, not just the cancer under study [17].

### 2.7. Conditional Survival

It is the probability of a patient surviving for a specific time, given that the patient has already survived for a particular time [18]. Conditional survival provides insight into how prognosis improves for patients who have already survived a certain period after diagnosis. We calculated conditional survival of four years for those who survived for one year, six years for those who survived for three years, and eight years for those who survived for five years.

Regarding mortality outcomes, we calculated standardized mortality ratios (SMRs), which are used to compare the mortality rate of a study population to the mortality rate of a standard or general population by dividing the number of invasive ependymoma deaths observed by the number of deaths predicted by the age- and sex-matched population in the same period to ensure comparability between groups. SMRs are used to compare the study population’s mortality rate to the general population’s mortality rate [19]. Moreover, our survival and mortality outcomes were stratified by age (<18, 18–44, 45–59, 60–74, >75), sex, and race (black, white, American Indian/Alaska Native, and Isan or pacific islander).

## 3. Results

### 3.1. Baseline Characteristics

This study included a total of 3821 patients. The study sample comprised 1960 males (51%) and 1861 females (49%). The largest proportion of patients (1269, 33%) was between 18 and 44 years of age. White patients made up the majority of the patient population (3153, 83%), and the most common ependymoma site was the spinal cord (C72.0) (1649 patients, 43%). Histologically, most of the tumors were diagnosed as ependymoma, not otherwise specified (NOS) (2980 patients, 78%), and anaplastic ependymoma accounted for a lower proportion (738 patients, 19%) [Table 1].

### 3.2. Mortality Rate and Causes of Death

This study included a total of 3821 patients, with 842 (22%) deaths [Table 2]. The highest mortality rate (244 deaths, 29%) was seen in pediatric patients younger than 18. Most of the deaths (688 deaths, 83%) were white people. Patients with cancers in the spinal cord had the greatest mortality rate (229 deaths, 43%). Patients with ependymoma NOS accounted for the majority of histological deaths (572 deaths, 78%). Among the total deaths, 424 (52%) resulted from brain and other nervous system cancers, 75 (9%) from non-CNS cancers, and 321 (39%) from non-cancer causes. The most common non-cancer causes included cerebrovascular diseases, pneumonia, and septicemia, with respective SMRs of 2.84 (95% CI, 1.86–4.17), 3.05 (95% CI, 1.52–5.45), and 3.35 (95% CI, 1.61–6.61).

### 3.3. Causes of Death Within One Year of the Diagnosis

A total of 198 deaths, representing 24.1% of all deaths, occurred within one year following the diagnosis of invasive ependymoma. Among these, 102 patients (52%) died of brain and other nervous system cancers, 13 patients (7%) died from non-CNS cancers, and 83 patients (42%) died due to non-cancer causes. Cerebrovascular diseases (seven deaths; SMR, 8.76; 95% CI, 3.52–18.05) were the most prevalent non-cancer causes, followed by infectious and parasitic diseases (five deaths; SMR, 17.57; 95% CI, 5.71–41.01). Patients under 18 who were diagnosed with invasive ependymoma had a greater risk of mortality within one year compared to those without the disease at the same age (SMR, 54.77; 95% CI, 38.95–74.88). Non-cancer causes were responsible for 30% of deaths among patients under the age of 18 and for 48% of deaths among patients aged 18 to 44 within one year following their diagnosis [Supplementary Table S2].

### 3.4. Causes of Death from One to Five Years After the Diagnosis

Between one and five years after being diagnosed with invasive ependymoma, 342 patients died. Among them, 211 patients (62%) died of brain and other nervous system malignancies, 30 (9%) died of non-CNS cancers, and 101 (30%) died of non-cancer causes. Cerebrovascular diseases (nine deaths, SMR, 3.18; 95% CI, 1.46–6.04) were the most common non-cancer causes. Brain and other nervous system cancers accounted for 82% of mortality in patients under 18 between one and five years following diagnosis.

### 3.5. Causes of Death After Five Years Following the Diagnosis

A total of 280 patients died over five years after being diagnosed with invasive ependymoma. Among them, 111 patients (40%) died of brain and other nervous system cancers, 32 (11%) died of non-CNS cancers, and 137 (49%) died of non-cancer causes. In patients aged 60–74 years, cerebrovascular disorders (eight deaths, SMR, 3.27; 95% CI, 1.41–6.45) were the most frequent non-cancer causes after five years following invasive ependymoma diagnosis.

### 3.6. Overall Survival Rate

In total, the observed survival rates at one, three, and five years were 94% (95% CI: 0.94–0.95), 88% (95%CI: 0.87–0.89), and 84% (95%CI: 0.82–0.85). For patients who survived the first year, three-year conditional survival was 91% (95% CI: 0.90–0.92), with a relative survival of 92% (95% CI: 0.91–0.93). For those who survived three years, three-year conditional survival was 94% (95% CI: 0.93–0.95), with a relative survival rate of 95% (95% CI: 0.94–0.96). For those who survived 5 years, 3-year conditional and relative survival rates were 94% (95%CI: 0.93–0.95) and 96% (95%CI: 0.95–0.97), respectively [Table 3].

### 3.7. Survival Rate According to Age, Sex and Race

At one, three, and five years, the observed survival rates were higher for females: 95% (95%CI: 0.94–0.96), 90% (95%CI: 0.88–0.91), and 86% (95%CI: 0.84–0.87), respectively. American Indian/Alaska Native patients had the lowest survival rates, with 91% (95%CI: 0.68–0.98), 86% (95%CI: 0.62–0.95), and 74% (95%CI: 0.48–0.89) observed survival at one, three, and five years. Patients aged 18–44 showed the highest observed survival rates of 97% (95%CI: 0.96–0.98), 94% (95%CI: 0.92–0.95), and 91% (95%CI: 0.89–0.93) [Table 4].

## 4. Discussion

Ependymoma is a neoplasm that arises from progenitor cells lining the ventricular system and the central canal of the spinal cord [9]. It is typically a benign tumor that arises from the ependymal cells of the central nervous system [20]. It represents 3–6% of all CNS tumors, and about 50% of them occur in the spine, although primary spinal cord tumors are relatively infrequent and represent only 4–10% of all CNS tumors [3,5,21,22,23].

The present study included 3821 patients, 51% (1960) of whom were males. The largest age group was those between 18 and 44 years old and represented 33% (1269) of the included population. Most of the patients were white (3153, 83%). Ryu et al., 2019, included a comparable sample, with males representing 53.5% and white people at 88.2% [3]. McGuire et al., 2009, reported that the incidence was higher among males and older adults above 40 years old [24]. Previous studies have reported increased male incidence compared to females [24,25,26,27]. A previous study conducted on patients from the US diagnosed with anaplastic ependymoma and ependymoma NOS in the period between 2000 and 2006 found that the incidence rate of both anaplastic ependymoma and ependymoma NOS was significantly higher in white people compared to all other races, except for anaplastic ependymoma, in which the incidence was not significantly higher in white people compared to Asia/Pacific Islanders, and in that study, the white population comprised 86.8% of the patients, which is comparable to this study [10]. The same study reported that the incidence of anaplastic ependymoma was not significantly different among males and females, while ependymoma NOS incidence was significantly higher in male patients [10]. According to this study, the most common ependymoma site was the spinal cord (C72.0) (1649 patients, 43%). This aligns with previous studies that reported ependymoma as the commonest primary intramedullary spinal cord tumor and that the spine represents 30–45% of the ependymoma site or about 50%, according to other studies [2,3,4,9,10]. Ryu et al., 2019, reported that 94.1% of all spine ependymomas were explicitly located in the spinal cord [3]. In pediatrics, about 90% of ependymomas are intracranial; in adults, about 60% are spinal, according to McGuire et al., 2009 [24].

The incidence of anaplastic ependymoma was the highest in the supratentorial location, followed by the infratentorial location, and lowest in the spinal cord, while ependymoma NOS was highest in the spinal cord, followed by the infratentorial and then the supratentorial locations [10].

The most common histological type was ependymoma NOS (2980 patients, 78%), followed by anaplastic ependymoma (738 patients, 19%). The annual percent change in the overall incidence of ependymoma increased by 2.1% from 2000 to 2010 [25]. In Ryu et al., 2019, which studied the spinal ependymomas, only 2.1% were anaplastic, while 65.1% of the cases were non-myxo papillary low grade and 32.8% were myxo papillary [3].

According to a previous study, the incidence of anaplastic ependymoma and ependymoma NOS was similar in the pediatric population aged 0–4 years, but, on the other hand, the ependymoma NOS had a significantly higher incidence in other pediatric age groups, 5–9 and 10–14 years [10]. The anaplastic-type incidence plateaued in adults and then declined in geriatrics above 85 years old. At the same time, ependymoma NOS peaked in the 45–69 age group and declined in those in the 80s [10]. This is consistent with previous reports on ependymoma incidence trends in the USA [9,25,26].

Of this study’s 3821 participants, 842 (22%) died. Ryu et al., 2019, reported a 10-year survival of 85% [3]. The 10-year survival of the spinal ependymomas was reported to be excellent, exceeding 90% [28,29,30]. A retrospective study conducted on patients treated for ependymoma between 1990 and 2009 found that the death rate was 15% during the follow-up period [31].

According to this study, the highest mortality rate was among patients younger than 18 (244 deaths, 29%), white (688 deaths, 83%), and those with cancer in the spinal cord (229 deaths, 43%). This could be aligned with the fact that 83% of this study population was white, and 43% of the disease was in the spinal cord.

The observed survival rates were higher among females; the patients aged 18–44 showed the highest observed survival rates, and American Indian/Alaska Native patients had the lowest survival rates.

A previous study reported that anaplastic ependymoma and ependymoma NOS had a 78% and 91% increased risk of death, respectively, in African Americans compared to the white population, which aligns with our findings in Supplementary Table S3 [10]. Another study reported that in gliomas, non-Hispanic white people had a lower survival rate than African Americans, contrary to our results [32]. A study reported that children had a worse prognosis when compared to adults [33]. In anaplastic ependymoma, adults older than 40 years had a 5-times greater risk of death compared to children aged 0–14 years, while in ependymoma NOS, young adults aged 15–39 years had a lower risk of death compared to children [10]. Supratentorial ependymoma and infratentorial ependymoma had 2.5- and 2.76-times greater risk of mortality than spinal cord ependymoma [10]. These results align with our findings, explaining the higher mortality rate in patients under 18, as approximately 90% of pediatric ependymomas are intracranial. In contrast, about 60% of adults are spinal, according to McGuire et al., 2009 [27]. The aggressive nature of the tumor in children may be due to the immaturity of neural tissue and unfavorable histopathological findings [20,34]. Many studies concluded that the patient’s gender is not a significant prognostic factor [35,36,37,38], while others have reported worse survival for female patients [39,40]. On the contrary, the results of the present study, in accordance with two previous studies, reported better survival for female patients [33,41].

Patients under 18 diagnosed with invasive ependymoma had a greater risk of mortality within one year compared to those without the disease at the same age (SMR, 54.77; 95% CI, 38.95–74.88). Within one year of diagnosis, the non-cancer causes were responsible for 30% of deaths of patients under 18 and 48% of patients aged 18–44. Brain and other nervous system cancers accounted for 82% of mortality in patients under 18 within one to five years following diagnosis, while in patients aged 60–74 years, cerebrovascular disorders (eight deaths, SMR, 3.27; 95% CI, 1.41–6.45) were the most frequent non-cancer causes after five years following invasive ependymoma diagnosis.

The most common pediatric solid malignancy is brain tumors, which are the leading cause of mortality related to cancers [42,43], with the ependymoma being the third most common pediatric brain tumor after astrocytoma and medulloblastoma, with over 50% arising before the age of 5 [44,45]. The very limited outcomes in younger patients may be due to the higher incidence of high-grade ependymoma and the higher rate of lateral and posterior fossa tumors, which infiltrate the nearby vital structures, complicating the gross total resection of the tumor and the mandatory delay of the radiotherapy below 3 years of age [31]. In adolescents and young adults, the mortality rate associated with cancer has declined by 0.8%, but survival rates with CNS tumors have not improved [46].

We found that ependymoma NOS accounted for 78% of all histological deaths. Previous studies found that the tumor site, histology, age, and sex are significant predictors of the patient’s survival [6,47,48,49]. A previous study reported that those with anaplastic ependymoma had a 2-times higher risk of death compared to ependymoma NOS [10].

The most frequent cause of death in this study was brain and other nervous system cancers, accounting for 52% of all deaths. The non-cancer causes accounted for 39% of all deaths, and 9% of deaths were due to non-CNS cancers.

We found that the predominant non-cancer causes were cerebrovascular diseases (26 deaths, SMR, 2.84; 95% CI, 1.86–4.17), pneumonia and influenza (11 deaths, SMR, 3.05; 95% CI, 1.52–5.45), septicemia (10 deaths, SMR, 3.35; 95% CI, 1.61–6.61), and other infectious and parasitic diseases (8 deaths, SMR, 2.99; 95% CI, 1.29–5.9). A study reported that in the last 4 weeks of life, 85% of patients with brain tumors presented with dysphagia [50]. Dysphagia associated with brain tumors can cause dehydration, undernutrition, and precipitate aspiration pneumonia [51].

In total, the observed survival rates in this study at one, three, and five years were 94%, 88%, and 84%, respectively. The 10-year survival of the spinal ependymomas was reported to be excellent, exceeding 90% [28,29,30].

In this study, 24.1% of deaths occurred within one year of diagnosis with invasive ependymoma, while 41.7% of deaths occurred from one to five years and 34.1% after five years. Brain and other nervous system cancers accounted for 52% of deaths within one year, 62% from one to five years, and 40% after five years. At the same time, non-cancer causes accounted for 42% of deaths within one year, 30% from one to five years, and 49% after five years. Further, 7% of deaths within one year, 9% from one to five years, and 11% after five years were the result of non-CNS cancers. Cerebrovascular diseases were the most common non-cancer causes of death within one year, from one to five years, and after five years of diagnosis with invasive ependymoma.

This study highlights the scarcity of evidence on the causes of death in ependymoma patients, making it a valuable starting point for addressing this topic. This will help move towards a more personalized healthcare approach in dealing with ependymoma patients, improving survival and decreasing mortality and morbidity. It also reveals the necessity for high-quality, dedicated research to address the causes of death in ependymoma patients.

This study has several limitations that should be considered when interpreting the findings. First, our analysis relies on the SEER database, which, while population-based and nationally representative, lacks detailed clinical information, such as the extent of surgical resection, radiation therapy parameters, chemotherapy regimens, molecular or genetic profiling, performance status, and comorbidities. The absence of these data limits our ability to adjust for key prognostic variables and may introduce residual confounding, particularly in subgroup comparisons across age, sex, and racial categories. Second, treatment details—especially the completeness of tumor resection and adjuvant therapy—are not consistently captured in SEER. Although the database includes some treatment variables (e.g., radiation), these are often incomplete or not applicable for CNS tumors, and there is no reliable coding for chemotherapy administration in many cases. As a result, we were unable to incorporate treatment modalities into our survival models, which may affect the generalizability and clinical interpretability of our results. Third, the causes of death in SEER data were derived from death certificates, which may lead to underreporting, especially for non-cancer causes, such as septicemia, cerebrovascular disease, and infections. While the use of SMRs helps contextualize excess mortality relative to the general population, inaccuracies in coding could influence the precision of these estimates. Fourth, despite the large sample size, some subgroup analyses—particularly for minority racial groups such as American Indian/Alaska Native and Asian/Pacific Islander populations—were based on small numbers, which may reduce statistical power and increase variability in survival estimates. Finally, the retrospective nature of the study design inherently limits causal inference. Unmeasured socioeconomic, access-to-care, and healthcare utilization factors may contribute to observed disparities in survival and should be explored in future prospective or linked healthcare database studies.

On the other hand, the relatively large sample size, being a population-based study from a high-quality database (SEER), which includes about 28% of the cancer patients in the USA and the independent, prospective collection of data, which decreases the recall bias, increases the credibility of our study.

## 5. Conclusions

The death rate was highest among pediatric patients under 18 years of age. Cerebrovascular disorders were the leading non-cancer cause of death across all time intervals. The probability of surviving for three years increases for patients who have already survived one, three, or five years post-diagnosis of invasive ependymoma. We present our findings to medical professionals to explore the potential development of future guidelines for the management of invasive ependymoma.

## Figures and Tables

**Table 1:** Baseline characteristics of patients with invasive ependymoma and mortality outcomes.

Characteristics	Diagnosed Cases No (%)	Deaths No (%)
All patients	3821 (100%)	842 (22%)
Age at diagnosis		
less than 18 years	1004 (26%)	244 (29%)
18–44	1269 (33%)	174 (21%)
45–59	949 (25%)	174 (21%)
60–75	504 (13%)	180 (21%)
more than 75	95 (2%)	70 (8%)
Sex		
Male	1960 (51%)	477 (51%)
Female	1861 (49%)	365 (49%)
Race		
American Indian/Alaska Native	22 (1%)	6 (1%)
Asian or Pacific Islander	252 (7%)	41 (7%)
white	3153 (83%)	688 (83%)
Black	344 (9%)	105 (9%)
Unknown	50 (1%)	2 (1%)
Primary site—labeled		
C41.2—Vertebral column	7 (0%)	2 (0%)
C48.0—Retroperitoneum	1 (0%)	-
C49.0—Connective, subcutaneous, other soft tissue: head, face, neck	1 (0%)	1 (0%)
C49.5—Connective, subcutaneous, other soft tissue: pelvis	3 (0%)	-
C49.6—Connective, subcutaneous, other soft tissue: trunk, NOS	2 (0%)	-
C56.9—Ovary	1 (0%)	-
C57.4—Uterine adnexa	1 (0%)	-
C70.0—Cerebral meninges	1 (0%)	-
C70.1—Spinal meninges	35 (1%)	5 (1%)
C70.9—Meninges, NOS	2 (0%)	-
C71.0—Cerebrum	39 (1%)	18 (1%)
C71.1—Frontal lobe	155 (4%)	46 (4%)
C71.2—Temporal lobe	70 (2%)	38 (2%)
C71.3—Parietal lobe	127 (3%)	37 (3%)
C71.4—Occipital lobe	59 (2%)	12 (2%)
C71.5—Ventricle, NOS	384 (10%)	99 (10%)
C71.6—Cerebellum, NOS	215 (6%)	75 (6%)
C71.7—Brain stem	610 (16%)	151 (16%)
C71.8—Overlapping lesion of the brain	107 (3%)	35 (3%)
C71.9—Brain, NOS	285 (7%)	82 (7%)
C72.0—Spinal cord	1649 (43%)	229 (43%)
C72.1—Cauda equina	51 (1%)	8 (1%)
C72.5—Cranial nerve, NOS	1 (0%)	-
C72.8—Overlapping lesion of brain & CNS	2 (0%)	-
C72.9—Nervous system, NOS	5 (0%)	1 (0%)
C75.3—Pineal gland	8 (0%)	3 (0%)
ICD-O-3 Histological Behavior, malignant		
Sub ependymoma, malignant	3 (0%)	-
Ependymoma NOS	2980 (78%)	572 (78%)
Ependymoma, anaplastic	738 (19%)	260 (19%)
Papillary ependymoma NOS	57 (1%)	7 (1%)
Myxopapillary ependymoma, malignant	35 (1%)	3 (1%)
Ependymoma, RELA fusion-positive	8 (0%)	-

**Table 2:** Standardized mortality ratios for causes of death following the diagnosis of invasive ependymoma.

Deaths by Time After Diagnosis								
	<1 Year		1–5 Years		>5 Years		Total	
	Observed No (%)	SMR (95% CI)	Observed No (%)	SMR (95% CI)	Observed No (%)	SMR (95% CI)	Observed No (%)	SMR (95% CI)
All Causes of Death	198 (100%)	10.54 (9.12–12.11)	342 (100%)	5.24 (4.7–5.83)	280 (100%)	2.28 (2.02–2.56)	820 (100%)	3.96 (3.69–4.24)
Brain and Other Nervous System	102 (52%)	655 (534–795)	211 (62%)	392 (341–449)	111 (40%)	114 (93.57–137)	424 (52%)	254 (230–279)
Non-CNS cancers	13 (7%)	2.66 (1.41–4.54)	30 (9%)	1.77 (1.19–2.52)	32 (11%)	1.02 (0.7–1.44)	75 (9%)	1.41 (1.11–1.76)
Non-cancer causes	83 (42%)	6.04 (4.81–7.49)	101 (30%)	2.12 (1.72–2.57)	137 (49%)	1.51 (1.27–1.79)	321 (39%)	2.11 (1.89–2.35)
Septicemia	4 (2%)	15.4 (4.2–39.42)	2 (1%)	2.17 (0.26–7.84)	4 (1%)	2.22 (0.6–5.67)	10 (1%)	3.35 (1.61–6.16)
Other Infectious and Parasitic Diseases, including HIV	5 (3%)	17.57 (5.71–41.01)	0 (0%)	0 (0–3.93)	3 (1%)	2.07 (0.43–6.05)	8 (1%)	2.99 (1.29–5.9)
Diabetes Mellitus	2 (1%)	3.37 (0.41–12.16)	3 (1%)	1.44 (0.3–4.21)	5 (2%)	1.26 (0.41–2.94)	10 (1%)	1.5 (0.72–2.77)
Alzheimer’s (ICD-9 and 10 only)			0 (0%)	0 (0–3.08)	6 (2%)	1.91 (0.7–4.16)	6 (1%)	1.3 (0.48–2.83)
Cardiovascular Diseases	8 (4%)	1.79 (0.77–3.52)	18 (5%)	1.14 (0.68–1.81)	32 (11%)	1.08 (0.74–1.52)	58 (7%)	1.16 (0.88–1.5)
Cerebrovascular Diseases	7 (4%)	8.76 (3.52–18.05)	9 (3%)	3.18 (1.46–6.04)	10 (4%)	1.81 (0.87–3.33)	26 (3%)	2.84 (1.86–4.17)
Pneumonia and Influenza	3 (2%)	9.43 (1.94–27.56)	4 (1%)	3.54 (0.96–9.06)	4 (1%)	1.85 (0.5–4.74)	11 (1%)	3.05 (1.52–5.45)
Chronic Obstructive Pulmonary Disease and Allied Cond	4 (2%)	4.04 (1.1–10.35)	5 (1%)	1.39 (0.45–3.25)	10 (4%)	1.34 (0.64–2.46)	19 (2%)	1.57 (0.95–2.46)
Chronic Liver Disease and Cirrhosis	1 (1%)	2.52 (0.06–14.05)	0 (0%)	0 (0–2.68)	1 (0%)	0.4 (0.01–2.21)	2 (0%)	0.47 (0.06–1.68)
Nephritis, Nephrotic Syndrome and Nephrosis	0 (0%)	0 (0–12.7)	1 (0%)	0.95 (0.02–5.3)	4 (1%)	1.92 (0.52–4.91)	5 (1%)	1.46 (0.47–3.41)
Accidents and Adverse Effects	0 (0%)	0 (0–2.88)	4 (1%)	0.94 (0.26–2.4)	10 (4%)	1.42 (0.68–2.61)	14 (2%)	1.11 (0.61–1.87)
Suicide and Self-inflicted Injury	1 (1%)	2.23 (0.06–12.4)	2 (1%)	1.33 (0.16–4.79)	1 (0%)	0.41 (0.01–2.31)	4 (0%)	0.92 (0.25–2.34)
Other Cause of Death	48 (24%)	14.44 (11–19.14)	53 (15%)	4.78 (3.58–6.25)	47 (17%)	2.21 (1.62–2.93)	148 (18%)	4.14 (3.5–4.87)

CNS: central nervous system; HIV: Human Immunodeficiency Virus; SMRs: standardized mortality ratios.

**Table 3:** Overall survival and survival according to gender and race.

Years	No of Patients	Observed Survival 95% CI (Lower-Upper)	Relative Survival 95% CI (Lower-Upper)
Overall			
1 Year	3821	94% (0.94–0.95)	95% (0.94–0.96)
3 years	3821	88% (0.87–0.89)	89% (0.88–0.9)
5 years	3821	84% (0.82–0.85)	86% (0.85–0.87)
3 years survival after 1 year survival	3374	91% (0.90–0.92)	92% (0.91–0.93)
3 years survival after 3 years survival	2741	94% (0.93–0.95)	95% (0.94–0.96)
3 years survival after 5 years survival	2281	94% (0.93–0.95)	96% (0.95–0.97)
Male			
1 Year	1960	94% (0.92–0.95)	94% (0.93–0.95)
3 Years	1960	86% (0.84–0.87)	85% (0.86–0.89)
5 Years	1960	82% (0.80–0.83)	84% (0.82–0.86)
3 years survival after 1 year survival	1716	89% (0.88–0.91)	91% (0.89–0.92)
3 years survival after 3 years survival	1383	93% (0.92–0.95)	95% (0.94–0.97)
3 years survival after 5 years survival	1142	93% (0.92–0.95)	95% (0.93–0.97)
Female			
1 Year	1861	95% (0.94–0.96)	96% (0.95–0.97)
3 Years	1861	90% (0.88–0.91)	91% (0.89–0.92)
5 Years	1861	86% (0.84–0.87)	88% (0.86–0.89)
3 years survival after 1 year survival	1658	92% (0.91–0.93)	93% (0.92–0.95)
3 years survival after 3 years survival	1358	94% (0.93–0.95)	95% (0.94–0.97)
3 years survival after 5 years survival	1139	95% (0.93–0.96)	97% (0.95–0.98)
White			
1 Year	3153	94% (0.94–0.95)	95% (0.94–0.96)
3 Years	3153	88% (0.87–0.89)	90% (0.88–0.91)
5 Years	3153	84% (0.83–0.86)	87% (0.85–0.88)
3 years survival after 1 year survival	2788	91% (0.90–0.92)	93% (0.91–0.94)
3 years survival after 3 years survival	2288	94% (0.93–0.95)	96% (0.94–0.96)
3 years survival after 5 years survival	1921	95% (0.93–0.956)	96% (0.95–0.97)
Black			
1 Year	344	93% (0.89–0.95)	93% (0.90–0.95)
3 Years	344	81% (0.76–0.85)	82% (0.77–0.86)
5 Years	344	77% (0.72–0.81)	79% (0.74–0.84)
3 years survival after 1 year survival	300	86% (0.81–0.89)	87% (0.82–0.91)
3 years survival after 3 years survival	234	93% (0.89–0.96)	95% (0.90–0.97)
3 years survival after 5 years survival	182	89% (0.83–0.93)	91% (0.84–0.95)
American Indian/Alaska Native			
1 Year	22	91% (0.68–0.98)	91% (0.67–0.98)
3 Years	22	86% (0.62–0.95)	86% (0.61–0.96)
5 Years	22	74% (0.48–0.89)	76% (0.49–0.91)
3 years survival after 1 year survival	18	88% (0.60–0.97)	89% (0.59–0.98)
3 years survival after 3 years survival	15	87% (0.56–0.97)	88% (0.55–0.97)
3 years survival after 5 years survival	13	92% (0.57–0.99)	93% (0.52–0.99)
Asian or Pacific Islander			
1 Year	252	96% (0.93–0.98)	96% (0.93–0.98)
3 Years	252	90% (0.85–0.93)	90% (0.86–0.94)
5 Years	252	85% (0.80–0.90)	86% (0.80–0.90)
3 years survival after 1 year survival	228	92% (0.87–0.95)	93% (0.88–0.96)
3 years survival after 3 years survival	176	94% (0.88–0.97)	94% (0.89–0.97)
3 years survival after 5 years survival	141	96% (0.91–0.98)	96% (0.91–0.99)

**Table 4:** Survival rate according to age.

Years	No of Patients	Observed Survival 95% CI (Lower–Upper)	Relative Survival 95% CI (Lower–Upper)
Age < 18			
1 Year	1004	96% (0.94–0.97)	96% (0.94–0.97)
3 Years	1004	85% (0.82–0.87)	85% (0.82–0.87)
5 Years	1004	78% (0.75–0.81)	78% (0.75–0.81)
3 years survival after 1 year survival	898	85% (0.82–0.87)	85% (0.82–0.87)
3 years survival after 3 years survival	676	89% (0.87–0.92)	90% (0.87–0.92)
3 years survival after 5 years survival	541	92% (0.89–0.94)	92% (0.89–0.94)
Age 18–44			
1 Year	1269	97% (0.96–0.98)	98% (0.97–0.98)
3 Years	1269	94% (0.92–0.95)	94% (0.92–0.95)
5 Years	1269	91% (0.89–0.93)	92% (0.90–0.93)
3 years survival after 1 year survival	1155	95% (0.93–0.96)	95% (0.93–0.96)
3 years survival after 3 years survival	982	96% (0.95–0.98)	97% (0.95–0.98)
3 years survival after 5 years survival	826	97% (0.96–0.98)	98% (0.96–0.99)
Age 45–59			
1 Year	949	95% (0.93–0.96)	96% (0.94–0.97)
3 Years	949	90% (0.88–0.92)	92% (0.90–0.94)
5 Years	949	88% (0.85–0.90)	90% (0.88–0.92)
3 years survival after 1 year survival	846	94% (0.92–0.95)	96% (0.94–0.97)
3 years survival after 3 years survival	717	96% (0.94–0.97)	98% (0.96–0.99)
3 years survival after 5 years survival	618	96% (0.94–0.98)	98% (0.96–0.99)
Age 60–74			
1 Year	484	89% (0.86–0.92)	90% (0.87–0.93)
3 Years	484	81% (0.77–0.85)	85% (0.81–0.89)
5 Years	484	78% (0.74–0.82)	84% (0.80–0.88)
3 years survival after 1 year survival	402	89% (0.85–0.92)	93% (0.89–0.96)
3 years survival after 3 years survival	318	94% (0.91–0.96)	99% (0.94–1)
3 years survival after 5 years survival	263	87% (0.82–0.91)	93% (0.87–0.96)
Age > 75			
1 Year	115	66% (0.57–0.74)	70% (0.60–0.78)
3 Years	115	52% (0.42–0.61)	62% (0.49–0.72)
5 Years	115	39% (0.30–0.49)	54% (0.40–0.67)
3 years survival after 1 year survival	73	66% (0.53–0.76)	80% (0.62_0.90)
3 years survival after 3 years survival	48	67% (0.51–0.78)	83% (0.57–0.94)
3 years survival after 5 years survival	33	65% (0.46–0.79)	85% (0.46–0.96)

## Data Availability

Data are available through the SEER Program (www.seer.cancer.gov, accessed on 31 January 2025).

## Supplementary Materials

The following supporting information can be downloaded at: https://www.mdpi.com/article/10.3390/medsci13030139/s1, Table S1: Definition of each cause of death and corresponding codes in the ICD-10 of Diseases and Related Health. Table S2. Shows the standardized mortality ratios (SMRs) for each cause of death following diagnosis of Invasive ependymoma according to age. Table S3. Survival of each age-specific group according to sex and race.

## Abbreviations

CNS	Central Nervous system
HIV	Human Immunodeficiency Virus
ICD-O	International Classification of Diseases for Oncology
NOS	Not otherwise specified
SEER	Surveillance Epidemiology and End Results
SMR	Standardized mortality ratio
