# Supplementary material for: Survival Patterns and Mortality Causes in Patients with Invasive Ependymoma: A Retrospective Cohort Analysis from 2000 to 2019
Source: Med Sci (Basel). 2025 Aug 16;13(3):139. doi: 10.3390/medsci13030139 (PMC12371993; doi:10.3390/medsci13030139)
Supplement: Supplementary file 1 [file medsci-13-00139-s001.zip › medsci-3738669-supplementary.pdf]

medical sciences

MDPI

Supplementary Table S1. Definition of each cause of death and corresponding codes in the ICD-10 of Diseases and Related Health.

Non-Cancer Causes of Death	ICD-10 corresponding codes	Cause of death definition
Septicemia	A40-A41	Sepsis
Other Infectious and Parasitic diseases including HIV	A00-A08	Intestinal infectious diseases
	A15-A19	Tuberculosis
	A20-A33	Certain zoonotic bacterial diseases
	A35-A39	Other bacterial diseases: tetanus, diphtheria, whooping cough, Scarlet fever, and meningococcal infection
	A42-A49	Other bacterial diseases: actinomycosis, nocardiosis, bartonellosis, erysipelas, and other bacterial infections of unspecified site
	A50-A53	Syphilis
	A54-B19	Infections with a predominantly sexual mode of transmission, other spirochaetal diseases, other diseases caused by chlamydiae, rickettsioses, viral infections of the central nervous system, arthropod-borne viral fevers, and viral hemorrhagic fevers, viral infections characterized by skin and mucous membrane lesions and viral hepatitis
	B25-B99	Other viral diseases, mycoses, protozoal diseases, helminthiases, pediculosis, acariasis, and other infestations, sequelae of infectious and parasitic diseases, bacterial, viral, and other infectious agents and diseases
Diabetes Mellitus	E10-E14	Diabetes mellitus
Alzheimer's	G30	Alzheimer disease
Cardiovascular Diseases	I00-I02	Acute rheumatic fever
	I05-I09	Chronic rheumatic heart diseases
	I11	Hypertensive heart disease
	I13	Hypertensive heart and renal disease
	I20-I25	Ischemic heart diseases
	I26-I28	Pulmonary heart disease and diseases of pulmonary circulation
	I30-I32	Diseases of pericardium
	I33	Acute and subacute endocarditis
	I34-I39	Nonrheumatic valve disorders
	I40-I41	Myocarditis
	I42-I43	Cardiomyopathy
	I44-I45	Conduction disorders
	I46	Cardiac arrest
	I47-I49	Arrythmias
	I50	Heart failure
	I51	Complications and ill-defined descriptions of heart disease
	I10	Essential (primary) hypertension
	I12	Hypertensive renal disease
	I70	Atherosclerosis
	I71	Aortic Aneurysm and Dissection
	I72-I73	Other aneurysms and dissection or other peripheral vascular diseases
	I74	Arterial embolism and thrombosis
	I77	Other disorders of arteries and arterioles
	I78	Diseases of capillaries
Cerebrovascular Diseases	I60-I62	Nontraumatic intracranial hemorrhage
	I63	Cerebral infarction
	I64	Stroke, not specified as hemorrhage or infarction
	I65-I66	Occlusion and stenosis of precerebral/cerebral arteries, not resulting in cerebral infarction
	I67-I69	Other cerebrovascular diseases or Sequelae of cerebrovascular disease
Pneumonia and Influenza	J09-J18	Influenza and pneumonia
Chronic Obstructive Pulmonary Disease	J40-J42	Bronchitis
	J43	Emphysema
	J44	Other chronic obstructive pulmonary disease
	J45-J46	Asthma or Status asthmaticus

	J47	Bronchiectasis
Chronic Liver Disease and Cirrhosis	K70	Alcoholic liver disease
	K73	Chronic hepatitis
	K74	Fibrosis and cirrhosis of liver
Nephritis, Nephrotic Syndrome and Nephrosis	N00-N07	Glomerular diseases
	N17-N19	Renal failure
	N25	Disorders resulting from impaired renal tubular function
	N26	Unspecified contracted kidney
	N27	Small kidney of unknown cause
Accidents and Adverse Effects	V01-V99	Transport accidents
	W00-X59	Other external causes of accidental injury
	Y85-Y86	Sequelae of transport accidents or other accidents
Suicide and Self-Inflicted Injury	X60-X84	Intentional self-harm
	Y87	Sequelae of intentional self-harm, assault and events of undetermined intent
Other Cause of Death	D00-D09	In situ neoplasms
	D10-D36	Benign neoplasms
	D37-D48	Neoplasms of uncertain or unknown behavior
	K25-K28	Stomach and Duodenal Ulcers
	A34	Complications of Pregnancy, Childbirth, Puerperium: Obstetrical tetanus
	O00-O95	Complications of Pregnancy, Childbirth, Puerperium: Pregnancy with abortive outcome, oedema, proteinuria and hypertensive disorders in pregnancy, childbirth and the puerperium, other maternal disorders predominantly related to pregnancy, maternal care related to the fetus and amniotic cavity and possible delivery problems and other complications of labor, delivery and the puerperium
	O98-O99	Complications of Pregnancy, Childbirth, Puerperium: Maternal infectious, parasitic and other diseases
	Q00-Q99	Congenital Anomalies
	R00-R99	Symptoms, signs, abnormal results of clinical or other investigative procedures, and ill-defined conditions regarding which no diagnosis classifiable elsewhere is recorded.
	P00-P96	Certain Conditions Originating in Perinatal Period: Fetus and newborn affected by maternal factors and by complications of pregnancy, labor and delivery, disorders related to length of gestation and fetal growth, birth trauma, respiratory and cardiovascular disorders specific to the perinatal period, infections specific to the perinatal period hemorrhagic and hematological disorders of fetus and newborn, transitory endocrine and metabolic disorders specific to fetus and newborn, digestive system disorders of fetus and newborn, conditions involving the integument and temperature regulation of fetus and newborn and other disorders originating in the perinatal period.
	X85-Y09	Homicide and Legal Intervention: Assault
	Y35	Legal Intervention
	Y87	Sequelae of assault
	Y89	Sequelae of legal intervention, war operations and unspecified external Cause

Supplementary Table S2. Shows the standardized mortality ratios (SMRs) for each cause of death following diagnosis of Invasive ependymoma according to age.

	Deaths by time after diagnosis							
	<1year		1-5 year		>5 years		Total	
	Observed No (%)	SMR (95% CI)	Observed No (%)	SMR (95% CI)	Observed No (%)	SMR (95% CI)	Observed No (%)	SMR (95% CI)
All Causes of Death	198(100%)	10.54(9.12-12.11)	342(100%)	5.24 (4.7-5.83)	280(100%)	2.28 (2.02-2.56)	820(100%)	3.96 (3.69-4.24)
Brain and Other Nervous System	102(52%)	655 (534 -795)	211(62%)	392 (341-449)	111(40%)	114 (93.57-137)	424(52%)	254 (230 -279)
Non-CNS cancers	13(7%)	2.66 (1.41-4.54)	30(9%)	1.77 (1.19-2.52)	32(11%)	1.02(0.7-1.44)	75(9%)	1.41 (1.11-1.76)
Non-cancer causes	83(42%)	6.04 (4.81-7.49)	101(30%)	2.12 (1.72-2.57)	137(49%)	1.51(1.27-1.79)	321(39%)	2.11 (1.89-2.35)

Septicemia	4(2%)	15.40 (4.2-39.42)	2(1%)	2.17(0.26-7.84)	4(1%)	2.22(0.6-5.67)	10(1%)	3.35 (1.61-6.16)
Other Infectious and Parasitic Diseases including HIV	5(3%)	17.57 (5.71-41.01)	0(0%)	0(0-3.93)	3(1%)	2.07(0.43-6.05)	8(1%)	2.99 (1.29-5.9)
Diabetes Mellitus	2(1%)	3.37(0.41-12.16)	3(1%)	1.44(0.3-4.21)	5(2%)	1.26(0.41-2.94)	10(1%)	1.5(0.72-2.77)
Alzheimer's (ICD-9 and 10 only)			0(0%)	0(0-3.08)	6(2%)	1.91(0.7-4.16)	6(1%)	1.3 (0.48-2.83)
Cardiovascular Diseases	8(4%)	1.79 (0.77-3.52)	18(5%)	1.14(0.68-1.81)	32(11%)	1.08(0.74-1.52)	58(7%)	1.16 (0.88-1.5)
Cerebrovascular Diseases	7(4%)	8.76 (3.52-18.05)	9(3%)	3.18(1.46-6.04)	10(4%)	1.81(0.87-3.33)	26(3%)	2.84 (1.86-4.17)
Pneumonia and Influenza	3(2%)	9.43 (1.94-27.56)	4(1%)	3.54(0.96-9.06)	4(1%)	1.85(0.5-4.74)	11(1%)	3.05 (1.52-5.45)
Chronic Obstructive Pulmonary Disease and Allied Cond	4(2%)	4.04 (1.1-10.35)	5(1%)	1.39(0.45-3.25)	10(4%)	1.34(0.64-2.46)	19(2%)	1.57(0.95-2.46)
Chronic Liver Disease and Cirrhosis	1(1%)	2.52 (0.06-14.05)	0(0%)	0(0-2.68)	1(0%)	0.4(0.01-2.21)	2(0%)	0.47(0.06-1.68)
Nephritis, Nephrotic Syndrome and Nephrosis	0(0%)	0(0-12.7)	1(0%)	0.95(0.02-5.3)	4(1%)	1.92(0.52-4.91)	5(1%)	1.46(0.47-3.41)
Accidents and Adverse Effects	0(0%)	0(0-2.88)	4(1%)	0.94(0.26-2.4)	10(4%)	1.42(0.68-2.61)	14(2%)	1.11(0.61-1.87)
Suicide and Self-Inflicted Injury	1(1%)	2.23(0.06-12.4)	2(1%)	1.33(0.16-4.79)	1(0%)	0.41(0.01-2.31)	4(0%)	0.92(0.25-2.34)
Other Cause of Death	48(24%)	14.44 (11-19.14)	53(15%)	4.78(3.58-6.25)	47(17%)	2.21 (1.62-2.93)	148(18%)	4.14 (3.5-4.87)
Age <18								
All Causes of Death	39(100%)	54.77 (38.95-74.88)	140(100%)	100 (84.21-118)	57(100%)	32.44 (24.57-42.03)	236(100%)	61.02 (53.48-69.32)
Brain and Other Nervous System	62%	3911 (2506-5819)	115(82%)	6073 (5014-7289)	50(88%)	2175 (1614 -2867)	189(80%)	3932(3392-4535)
Non-CNS cancers	5%	129(15.58-465)	9(6%)	189 (86.46-358.95)	1(2%)	11.1(0.28-61.83)	12(5%)	78.30 (40.46-137)
Non-cancer causes	33%	19(10.03-32.2)	16(11%)	12.01 (6.87-19.51)	6(11%)	3.65 (1.34-7.94)	35(15%)	9.55 (6.65-13.28)
Septicemia	5%	300 (36.37-1085)	0(0%)	0(0-318)	0(0%)	0(0-395)	2(1%)	72.49 (8.78-262)
Diabetes Mellitus	0%	0(0-5685)	0(0%)	0(0-1271)	1(2%)	73.45 (1.86-409)	1(0%)	58.26 (1.47-325)
Accidents and Adverse Effects	0%	0(0-37.06)	1(1%)	3.28(0.08-18.28)	0(0%)	0(0-4.95)	1(0%)	0.87(0.02-4.85)
Other Cause of Death	11(28%)	21(10.37-37.16)	15(11%)	17.56 (9.83-28.96)	5(9%)	11.19 (3.63-26.12)	31(13%)	16.93 (11.51-24.04)
Age 18-44								
All Causes of Death	32(100%)	19.06 (13.04-26.91)	67(100%)	10.34 (8.02-13.14)	69(100%)	4.35 (3.39-5.51)	168(100%)	7.00 (5.98-8.14)
Brain and Other Nervous System	16(50%)	893 (510 -1450)	44(66%)	630 (458-846)	22(32%)	122 (76.32-184)	82(49%)	306 (243-379)
Non-CNS cancers	1(3%)	4.88(0.12-27.19)	6(9%)	6.46 (2.37-14.06)	4(6%)	1.2(0.33-3.07)	11(7%)	2.46 (1.23-4.41)
Non-cancer causes	15(47%)	10.30 (5.77-17)	17(25%)	3.10 (1.81-4.97)	43(62%)	3.49 (2.52-4.69)	75(45%)	3.89 (3.06-4.88)
Septicemia			0(0%)	0(0-57.2)	1(1%)	4.99(0.13-27.82)	1(1%)	3.58(0.09-19.92)
Other Infectious and Parasitic Diseases including HIV	1(3%)	19.14(0.48-107)	0(0%)	0(0-19.6)	1(1%)	2.95(0.07-16.44)	2(1%)	3.45(0.42-12.47)
Diabetes Mellitus	0(0%)	0(0-107)	0(0%)	0(0-24.17)	1(1%)	2(0.05-11.17)	1(1%)	1.46(0.04-8.12)
Cardiovascular Diseases	0(0%)	0(0-17.65)	1(1%)	1.07(0.03-5.97)	6(9%)	2.03(0.74-4.42)	7(4%)	1.71(0.69-3.52)
Cerebrovascular Diseases	1(3%)	30.5(0.77-169.92)	3(4%)	20.93 (4.32-61.17)	1(1%)	2.34(0.06-13.02)	5(3%)	8.28 (2.69-19.32)
Chronic Obstructive Pulmonary Disease and Allied Cond	1(3%)	69.73 (1.77-389)	1(1%)	14.61(0.37-81.41)	3(4%)	8.09 (1.67-23.65)	5(3%)	11.03 (3.58-25.73)
Chronic Liver Disease and Cirrhosis	0(0%)	0(0-78.01)	0(0%)	0(0-16.96)	1(1%)	1.42(0.04-7.93)	1(1%)	1.03(0.03-5.76)
Nephritis, Nephrotic Syndrome and Nephrosis	0(0%)	0(0-309)	0(0%)	0(0-69.89)	4(6%)	23.66 (6.45-60.58)	4(2%)	17.11 (4.66-43.81)
Accidents and Adverse Effects	0(0%)	0(0-7.68)	2(3%)	1.21(0.15-4.38)	7(10%)	2.60 (1.04-5.35)	9(5%)	1.87(0.85-3.54)
Suicide and Self-Inflicted Injury	0(0%)	0(0-20.02)	2(3%)	3.15(0.38-11.38)	1(1%)	0.97(0.02-5.39)	3(2%)	1.62(0.33-4.73)
Other Cause of Death	12(38%)	33.65 (17.39-58.79)	8(12%)	6.15 (2.66-12.12)	17(25%)	6.21 (3.62-9.94)	37(22%)	8.42 (5.93-11.6)
Age 45-59								
All Causes of Death	41(100%)	8.93 (6.41-12.11)	63(100%)	3.49(2.68-4.46)	68(100%)	1.49(1.16-1.89)	172(100%)	2.52 (2.16-2.93)
Brain and Other Nervous System	23(56%)	436 (276-654)	32(51%)	156.99(107.38-221.62)	24(35%)	50.54(32.38-75.2)	79(46%)	108.00 (85.51-134.6)
Non-CNS cancers	2(5%)	1.51(0.18-5.44)	6(10%)	1.1(0.4-2.39)	14(21%)	0.98(0.54-1.64)	22(13%)	1.04(0.65-1.58)
Non-cancer causes	16(39%)	4.98 (2.85-8.09)	25(40%)	2.02(1.31-2.98)	30(44%)	0.98(0.66-1.39)	71(41%)	1.53 (1.2-1.93)
Septicemia	1(2%)	16.32(0.41-90.93)	0(0%)	0(0-14.53)	0(0%)	0(0-5.24)	1(1%)	0.98(0.02-5.47)
Other Infectious and Parasitic Diseases including HIV	2(5%)	16.18 (1.96-58.43)	0(0%)	0(0-8.78)	0(0%)	0(0-5.44)	2(1%)	1.64(0.2-5.91)
Diabetes Mellitus	0(0%)	0(0-23.1)	1(2%)	1.53(0.04-8.52)	0(0%)	0(0-2.11)	1(1%)	0.39(0.01-2.18)
Alzheimer's (ICD-9 and 10 only)	0(0%)	0(0-686)		0(0-113.98)	1(1%)	3.08(0.08-17.17)	1(1%)	2.76(0.07-15.38)
Cardiovascular Diseases	3(7%)	2.89(0.6-8.45)	5(8%)	1.21(0.39-2.81)	10(15%)	0.94(0.45-1.72)	18(10%)	1.13(0.67-1.79)

Cerebrovascular Diseases	1(2%)	6.75(0.17-37.63)	3(5%)	5.09 (1.05-14.87)	1(1%)	0.61(0.02-3.38)	5(3%)	2.1(0.68-4.89)
Pneumonia and Influenza	2(5%)	35.94 (4.35-129.83)	1(2%)	4.42(0.11-24.6)	4(6%)	6.04(1.65-15.47)	7(4%)	7.41 (2.98-15.28)
Chronic Obstructive Pulmonary Disease and Allied Cond	0(0%)	0(0-25.12)	2(3%)	2.84(0.34-10.27)	2(3%)	0.74(0.09-2.67)	4(2%)	1.13(0.31-2.88)
Nephritis, Nephrotic Syndrome and Nephrosis	0(0%)	0(0-66.46)	1(2%)	4.24(0.11-23.64)	0(0%)	0(0-5.13)	1(1%)	0.99(0.03-5.51)
Accidents and Adverse Effects	0(0%)	0(0-9.41)	1(2%)	0.76(0.02-4.24)	2(3%)	0.96(0.12-3.47)	3(2%)	0.79(0.16-2.32)
Other Cause of Death	7(17%)	10.53 (4.23-21.69)	11(17%)	4.31 (2.15-7.71)	10(15%)	1.51(0.72-2.78)	28(16%)	2.84 (1.89-4.11)
Age 60-74								
All Causes of Death	50(100%)	7.28 (5.4-9.6)	45(100%)	1.851.35-2.48)	66(100%)	1.46 (1.13-1.86)	161(100%)	2.11 (1.79-2.46)
Brain and Other Nervous System	25(50%)	407.24 (264-601)	16(36%)	78.86 (45.08-128.07)	10(15%)	37.19 (17.83-68.39)	51(32%)	95.66 (71.22-125.77)
Non-CNS cancers	3(6%)	1.31(0.27-3.82)	5(11%)	0.64(0.21-1.5)	10(15%)	0.86(0.41-1.58)	18(11%)	0.83(0.49-1.31)
Non-cancer causes	22(44%)	4.88 (3.06-7.39)	24(53%)	1.47(0.94-2.19)	46(70%)	1.38 (1.01-1.84)	92(57%)	1.70 (1.37-2.08)
Septicemia	0(0%)	0(0-35.19)	2(4%)	5.28(0.64-19.08)	2(3%)	2.87(0.35-10.35)	4(2%)	3.39(0.92-8.67)
Other Infectious and Parasitic Diseases including HIV	1(2%)	14.15(0.36-78.83)	0(0%)	0(0-16.45)	2(3%)	5.95(0.72-21.48)	3(2%)	4.75(0.98-13.89)
Diabetes Mellitus	2(4%)	7.62 (0.92-27.54)	1(2%)	1.12(0.03-6.24)	2(3%)	1.43(0.17-5.16)	5(3%)	1.96(0.64-4.56)
Alzheimer's (ICD-9 and 10 only)	0(0%)	0(0-59.8)	0(0%)	0(0-10.5)	4(6%)	2.26(0.62-5.79)	4(2%)	1.83(0.5-4.69)
Cardiovascular Diseases	4(8%)	2.33 (0.63-5.96)	5(11%)	0.84(0.27-1.96)	10(15%)	0.88(0.42-1.62)	19(12%)	1(0.6-1.56)
Cerebrovascular Diseases	4(8%)	13.96 (3.8-35.75)	3(7%)	2.82(0.58-8.23)	8(12%)	3.27 (1.41-6.45)	15(9%)	3.95 (2.21-6.52)
Pneumonia and Influenza	0(0%)	0(0-35.33)	1(2%)	2.54(0.06-14.15)	0(0%)	0(0-4.2)	1(1%)	0.73(0.02-4.05)
Chronic Obstructive Pulmonary Disease and Allied Cond	2(4%)	4.17(0.5-15.06)	1(2%)	0.54(0.01-3.01)	5(8%)	1.4(0.45-3.26)	8(5%)	1.35(0.58-2.67)
Chronic Liver Disease and Cirrhosis	1(2%)	7.71(0.2-42.98)	0(0%)	0(0-9.48)	0(0%)	0(0-8.43)	1(1%)	1.05(0.03-5.83)
Accidents and Adverse Effects	0(0%)	0(0-18.94)	0(0%)	0(0-5.74)	1(2%)	0.88(0.02-4.89)	1(1%)	0.51(0.01-2.82)
Other Cause of Death	8(16%)	8.80 (3.8-17.35)	11(24%)	3.15 (1.57-5.64)	12(18%)	1.46(0.76-2.55)	31(19%)	2.46 (1.67-3.49)
Age >75								
All Causes of Death	36(100%)	7.28 (5.1-10.08)	27(100%)	1.80 (1.19-2.62)	20(100%)	1.36(0.83-2.11)	83(100%)	2.40 (1.91-2.97)
Brain and Other Nervous System	14(39%)	800.41 (437.59-1342.96)	4(15%)	94.71 (25.81-243)	5(25%)	175.37 (56.94-409)	23(28%)	261(165 -391)
Non-CNS cancers	5(14%)	4.76 (1.55-11.11)	4(15%)	1.46(0.4-3.74)	3(15%)	1.43(0.29-4.18)	12(14%)	2.04 (1.05-3.56)
Non-cancer causes	17(47%)	4.38 (2.55-7.02)	19(70%)	1.55(0.94-2.43)	12(60%)	0.96(0.49-1.67)	48(58%)	1.68 (1.24-2.22)
Septicemia	1(3%)	13.87(0.35-77.29)	0(0%)	0(0-17.33)	1(5%)	5.15(0.13-28.67)	2(2%)	4.17(0.51-15.07)
Other Infectious and Parasitic Diseases including HIV	1(3%)	32.41(0.82-180.6)	0(0%)	0(0-40.08)	0(0%)	0(0-44.73)	1(1%)	4.87(0.12-27.13)
Diabetes Mellitus	0(0%)	0(0-26.88)	1(4%)	2.64(0.07-14.72)	1(5%)	3.18(0.08-17.72)	2(2%)	2.41(0.29-8.7)
Alzheimer's (ICD-9 and 10 only)	0(0%)	0(0-17.38)	0(0%)	0(0-4.53)	1(5%)	0.97(0.02-5.4)	1(1%)	0.49(0.01-2.71)
Cardiovascular Diseases	1(3%)	0.67(0.02-3.72)	7(26%)	1.5(0.6-3.09)	6(30%)	1.29(0.47-2.81)	14(17%)	1.3(0.71-2.17)
Cerebrovascular Diseases	1(3%)	3.06(0.08-17.03)	0(0%)	0(0-3.62)	0(0%)	0(0-3.76)	1(1%)	0.43(0.01-2.39)
Pneumonia and Influenza	1(3%)	7.53(0.19-41.97)	2(7%)	4.71(0.57-17.02)	0(0%)	0(0-8.99)	3(4%)	3.1(0.64-9.06)
Chronic Obstructive Pulmonary Disease and Allied Cond	1(3%)	2.89(0.07-16.12)	1(4%)	1.05(0.03-5.83)	0(0%)	0(0-4.47)	2(2%)	0.94 (0.11-3.4)
Suicide and Self-Inflicted Injury	1(3%)	59.56 (1.51-332)	0(0%)	0(0-89.54)	0(0%)	0(0-113.73)	1(1%)	11.06 (0.28-61.62)
Other Cause of Death	10(28%)	11.56 (5.54-21.26)	8(30%)	2.77 (1.19-5.45)	3(15%)	0.91(0.19-2.67)	21(25%)	2.98 (1.85-4.56)

Supplementary Table S3. Survival of each age-specific group according to sex and race.

Years	No of Patients	Observed Survival 95% CI (lower-upper)	Relative Survival 95% CI (lower- upper)
Age<18			
1 Year	1004	96% (0.94_0.97)	96% (0.94-0.97)
3 Years	1004	85% (0.82_0.87)	85% (0.82-0.87)
5 Years	1004	78% (0.75_0.81)	78% (0.75-0.81)
3 years survival after 1 year survival	898	85% (0.82_0.87)	85% (0.82-0.87)

3 years survival after 3 years survival	676	89% (0.87_0.92)	90% (0.87-0.92)
3 years survival after 5 years survival	541	92% (0.89_0.94)	92% (0.89-0.94)
Male			
1 Year	527	96% (0.94-0.97)	96% (0.94-0.97)
3 Years	527	84% (0.80-0.87)	84% (0.8-0.87)
5 Years	527	76% (0.71-0.79)	76% (0.71-0.80)
3 years survival after 1 year survival	473	82% (0.78-0.85)	82% (0.78-0.85)
3 years survival after 3 years survival	351	87% (0.83-0.90)	87% (0.83-0.91)
3 years survival after 5 years survival	273	90% (0.86-0.93)	91% (0.86-0.94)
Female			
1 Year	477	96% (0.93-0.97)	96% (0.93-0.97)
3 Years	477	86% (0.82-0.89)	86% (0.82-0.89)
5 Years	477	81% (0.77-0.85)	81% (0.77-0.85)
3 years survival after 1 year survival	425	89% (0.85-0.91)	89% (0.85-0.91)
3 years survival after 3 years survival	325	92% (0.88-0.95)	92% (0.88-0.95)
3 years survival after 5 years survival	268	94% (0.90-0.96)	94% (0.90-0.96)
White			
1 Year	791	96% (0.94-0.97)	96% (0.94-0.97)
3 Years	791	86% (0.84-0.89)	86% (0.84-0.89)
5 Years	791	80% (0.77-0.83)	80% (0.77-0.83)
3 years survival after 1 year survival	708	86% (0.83-0.89)	86% (0.84-0.89)
3 years survival after 3 years survival	541	89% (0.86-0.92)	89% (0.86-0.92)
3 years survival after 5 years survival	437	92% (0.89-0.94)	92% (0.89-0.94)
Black			
1 Year	120	94% (0.88-0.97)	94% (0.88-0.97)
3 Years	120	72% (0.62-0.79)	72% (0.62-0.79)
5 Years	120	67% (0.57-0.75)	67% (0.57-0.75)
3 years survival after 1 year survival	107	74% (0.64-0.81)	74% (0.64-0.81)
3 years survival after 3 years survival	71	94% (0.84-0.98)	94% (0.84-0.98)
3 years survival after 5 years survival	55	90% (0.78-0.96)	90% (0.78-0.96)
American Indian/Alaska Native			
1 Year	8	88% (0.39-0.98)	88% (0.39-0.98)
3 Years	8	70% (0.23-0.92)	70% (0.22-0.92)
5 Years	8	53% (0.12-0.82)	53% (0.12-0.82)

3 years survival after 1 year survival	5	80% (0.20-0.97)	80% (0.20-0.97)
3 years survival after 3 years survival	4	75% (0.13-0.96)	75% (0.13-0.96)
3 years survival after 5 years survival	3	-	-
Asian or Pacific islander			
1 year	74	99% (0.91-100)	99% (0.91-100)
3 years	74	89% (0.79-0.95)	89% (0.79-0.95)
5 Years		78% (0.65-0.87)	78% (0.65-0.87)
3 years survival after 1 year survival	74	87% (0.76-0.93)	87% (0.76-0.93)
3 years survival after 3 years survival	70	87% (0.74-0.94)	87% (0.74-0.94)
3 years survival after 5 years survival	52	97% (0.80-100)	97% (0.80-100)
Age 18-44			
1 Year	1269	97% (0.96-0.98)	98% (0.97-0.98)
3 Years	1269	94% (0.92-0.95)	94% (0.92-0.95)
5 Years	1269	91% (0.89-0.93)	92% (0.90-0.93)
3 years survival after 1 year survival	1155	95% (0.93-0.96)	95% (0.93-0.96)
3 years survival after 3 years survival	982	96% (0.95-0.98)	97% (0.95-0.98)
3 years survival after 5 years survival	826	97% (0.96-0.98)	98% (0.96-0.99)
Male			
1 Year	646	97% (0.95-0.98)	97% (0.95-0.98)
3 Years	646	93% (0.90-0.94)	93% (0.91-0.95)
5 Years	646	90% (0.87-0.92)	91% (0.88-0.93)
3 years survival after 1 year survival	584	94% (0.92-0.96)	95% (0.92-0.96)
3 years survival after 3 years survival	493	96% (0.94-0.98)	97% (0.95-0.98)
3 years survival after 5 years survival	410	97% (0.95-0.98)	98% (0.95-0.99)
Female			
1 Year	623	98% (0.97-0.99)	98% (0.97-0.99)
3 Years	623	94% (0.92-0.96)	95% (0.92-0.96)
5 Years	623	92% (0.89-0.94)	92% (0.90-0.94)
3 years survival after 1 year survival	571	95% (0.93-0.97)	95% (0.93-0.97)
3 years survival after 3 years survival	489	96% (0.94-0.97)	96% (0.94-0.98)
3 years survival after 5 years survival	416	98% (0.96-0.99)	98% (0.96-0.99)
White			
1 Year	1035	97% (0.96-0.98)	97% (0.96-0.98)
3 Years	1035	93% (0.91-0.95)	94% (0.92-0.95)

5 Years	1035	91% (0.89-0.92)	91% (0.89-0.93)
3 years survival after 1 year survival	939	95% (0.93-0.96)	95% (0.93-0.96)
3 years survival after 3 years survival	802	97% (0.95-0.98)	97% (0.95-0.98)
3 years survival after 5 years survival	680	98% (0.96-0.99)	98% (0.97-0.99)
Black			
1 Year	112	97% (0.92-0.99)	97% (0.92-0.99)
3 Years	112	93% (0.87-0.97)	94% (0.87-0.97)
5 Years	112	90% (0.82-0.95)	91% (0.83-0.95)
3 years survival after 1 year survival	103	95% (0.88-0.98)	95% (0.88-0.98)
3 years survival after 3 years survival	90	94% (0.85-0.97)	94% (0.86-0.98)
3 years survival after 5 years survival	71	92% (0.83-0.97)	93% (0.83-0.97)
American Indian/Alaska Native			
1 Year	5	-	-
3 Years	5	-	-
5 Years	5	67% (0.05-0.95)	67% (0.05-0.95)
3 years survival after 1 year survival	5	67% (0.05-0.95)	67% (0.05-0.95)
3 years survival after 3 years survival	3	67% (0.05-0.95)	67% (0.05-0.95)
3 years survival after 5 years survival	2	-	-
Asian or Pacific islander			
1 Year	96	99% (0.93-1)	99% (0.92-1)
3 Years	96	97% (0.89-0.99)	97% (0.89-0.99)
5 Years	96	95% (0.88-0.98)	95 % (0.88-0.98)
3 years survival after 1 year survival	90	96% (0.89-0.99)	96% (0.88-0.99)
3 years survival after 3 years survival	75	95% (0.86-0.99)	96% (0.86-0.99)
3 years survival after 5 years survival	62	97% (0.87-0.99)	97% (0.87-0.99)
Age 45-59			
1 Year	949	95% (0.93-0.96)	96% (0.94-0.97)
3 Years	949	90% (0.88-0.92)	92% (0.90-0.94)
5 Years	949	88% (0.85-0.90)	90% (0.88-0.92)
3 years survival after 1 year survival	846	94% (0.92-0.95)	96% (0.94-0.97)
3 years survival after 3 years survival	717	96% (0.94-0.97)	98% (0.96-0.99)
3 years survival after 5 years survival	618	96% (0.94-0.98)	98% (0.96-0.99)
Male			

1 Year	480	94% (0.91-0.96)	95% (0.92-0.96)
3 Years	480	89% (0.86-0.92)	91% (0.87-0.93)
5 Years	480	86% (0.83-0.90)	89% (0.85-0.92)
3 years survival after 1 year survival	420	94% (0.91-0.96)	96% (0.92-0.97)
3 years survival after 3 years survival	362	96% (0.93-0.98)	98% (0.94-1)
3 years survival after 5 years survival	313	96% (0.93-0.98)	99% (0.92-1)
Female			
1 Year	469	96% (0.94-0.98)	96% (0.94-0.98)
3 Years	469	92% (0.89-0.94)	93% (0.90-0.95)
5 Years	469	89% (0.86-0.92)	91% (0.87-0.94)
3 years survival after 1 year survival	426	94% (0.91-0.96)	95% (0.92-0.97)
3 years survival after 3 years survival	355	96% (0.93-0.98)	97% (0.94-0.99)
3 years survival after 5 years survival	305	96% (0.93-0.98)	98% (0.94-0.99)
White			
1 Year	800	95% (0.93-0.96)	96% (0.94-0.97)
3 Years	800	91% (0.89-0.93)	92% (0.90-0.94)
5 Years	800	88% (0.86-0.90)	91% (0.88-0.93)
3 years survival after 1 year survival	716	94% (0.92-0.96)	96% (0.94-0.97)
3 years survival after 3 years survival	613	96% (0.94-0.97)	98% (0.96-0.99)
3 years survival after 5 years survival	533	97% (0.95-0.98)	99% (0.95-1)
Black			
1 Year	73	95% (0.86-0.98)	95% (0.86-0.98)
3 Years	73	86% (0.75-0.92)	88% (0.76-0.94)
5 Years	73	81% (0.69-0.88)	84% (0.71-0.91)
3 years survival after 1 year survival	65	89% (0.78-0.95)	91% (0.79-0.96)
3 years survival after 3 years survival	55	92% (0.795-0.97)	94% (0.78-0.99)
3 years survival after 5 years survival	43	83% (0.67-0.92)	86% (0.67-0.945)
American Indian/Alaska Native			
1 Year	6	-	-
3 Years	6	-	-
5 Years	6	-	-
3 years survival after 1 year survival	6	-	-
3 years survival after 3 years survival	6	-	-
3 years survival after 5 years survival	6	83% (0.27-0.98)	84% (0.25-0.98)
Asian or Pacific islander			

1 Year	59	93% (0.82-0.97)	93% (0.82-0.97)
3 Years	59	87% (0.74-0.94)	88% (0.75-0.94)
5 Years	59	87% (0.74-0.94)	88% (0.75-0.94)
3 years survival after 1 year survival	50	94% (0.81-0.98)	94% (0.81-0.98)
3 years survival after 3 years survival	38	-	-
3 years survival after 5 years survival	32	-	-
Age 60-74			
1 Year	484	89% (0.86-0.92)	90% (0.87-0.93)
3 Years	484	81% (0.77-0.85)	85% (0.81-0.89)
5 Years	484	78% (0.74-0.82)	84% (0.80-0.88)
3 years survival after 1 year survival	402	89% (0.85-0.92)	93% (0.89-0.96)
3 years survival after 3 years survival	318	94% (0.91-0.96)	99% (0.94-1)
3 years survival after 5 years survival	263	87% (0.82-0.91)	93% (0.87-0.96)
Male			
1 Year	246	86% (0.81-0.90)	88% (0.83-0.92)
3 Years	246	77% (0.71-0.82)	82% (0.75-0.87)
5 Years	246	75% (0.69-0.80)	81% (0.74-0.86)
3 years survival after 1 year survival	203	87% (0.81-0.91)	92% (0.86-0.96)
3 years survival after 3 years survival	156	95% (0.90-0.98)	99% (0.86-1)
3 years survival after 5 years survival	129	85% (0.76-0.90)	91% (0.81-0.96)
Female			
1 Year	238	92% (0.87-0.95)	93% (0.88-0.96)
3 Years	238	86% (0.80-0.90)	89% (0.83-0.92)
5 Years	238	82% (0.76-0.87)	88% (0.81-0.92)
3 years survival after 1 year survival	199	91% (0.86-0.94)	95% (0.88-0.98)
3 years survival after 3 years survival	162	93% (0.88-0.96)	98% (0.87-1)
3 years survival after 5 years survival	134	90% (0.83-0.94)	94% (0.85-0.98)
White			
1 Year	422	91% (0.88-0.93)	92% (0.89-0.95)
3 Years	422	83% (0.79-0.86)	87% (0.82-0.90)
5 Years	422	80% (0.75-0.83)	86% (0.81-0.90)
3 years survival after 1 year survival	360	89% (0.85-0.92)	93% (0.89-0.96)
3 years survival after 3 years survival	287	94% (0.90-0.96)	99% (0.94-1)
3 years survival after 5 years survival	240	88% (0.83-0.92)	94% (0.87-0.97)
Black			

1 Year	35	71% (0.52-0.83)	72% (0.53-0.85)
3 Years	35	67% (0.49-0.81)	69% (0.50-0.83)
5 Years	35	67% (0.49-0.81)	69% (0.50-0.83)
3 years survival after 1 year survival	22	95% (0.70-0.99)	96% (0.66-1)
3 years survival after 3 years survival	16	90% (0.47-0.99)	96% (0.8-1)
3 years survival after 5 years survival	11	80% (0.41-0.95)	83% (0.38-0.96)
American Indian/Alaska Native			
1 Year	3	67% (0.05-0.95)	67% (0.05-0.95)
3 Years	3	67% (0.05-0.95)	67% (0.05-0.95)
5 Years	3	67% (0.05-0.95)	67% (0.05-0.95)
3 years survival after 1 year survival	2	-	-
3 years survival after 3 years survival	2	-	-
3 years survival after 5 years survival	2	-	-
Asian or Pacific islander			
1 Year	19	84% (0.58-0.95)	84% (0.57-0.95)
3 Years	19	71% (0.44-0.87)	72% (0.44-0.88)
5 Years	19	71% (0.44-0.87)	72% (0.44-0.88)
3 years survival after 1 year survival	15	85% (0.52-0.96)	86% (0.52-0.97)
3 years survival after 3 years survival	10	-	-
3 years survival after 5 years survival	8	43% (0.01-0.85)	44% (0.01-0.86)
Age >75			
1 Year	115	66% (0.57-0.74)	70% (0.60-0.78)
3 Years	115	52% (0.42-0.61)	62% (0.49-0.72)
5 Years	115	39% (0.30-0.49)	54% (0.40-0.67)
3 years survival after 1 year survival	73	66% (0.53-0.76)	80% (0.62_0.90)
3 years survival after 3 years survival	48	67% (0.51-0.78)	83% (0.57-0.94)
3 years survival after 5 years survival	33	65% (0.46-0.79)	85% (0.46-0.96)
Male			
1 Year	61	62% (0.49-0.73)	66% (0.51-0.77)
3 Years	61	43% (0.30-0.56)	53% (0.36-0.67)
5 Years	61	37% (0.24-0.50)	52% (0.33-0.67)
3 years survival after 1 year survival	36	63% (0.44-0.77)	78% (0.48-0.92)
3 years survival after 3 years survival	21	76% (0.51-0.89)	94% (0.03-1)
3 years survival after 5 years survival	17	58% (0.31-0.77)	75% (0.29-0.94)
Female			

1 Year	54	71% (0.57-0.82)	75% (0.59-0.85)
3 Years	54	61% (0.47-0.73)	68% (0.52-0.80)
5 Years	54	42% (0.28-0.56)	55% (0.35-0.72)
3 years survival after 1 year survival	37	69% (0.50-0.82)	80% (0.53-0.92)
3 years survival after 3 years survival	27	59% (0.37-0.76)	73% (0.41-0.90)
3 years survival after 5 years survival	16	73% (0.44-0.89)	92% (0.04-1)
White			
1 Year	105	65% (0.55-0.73)	69% (0.58-0.77)
3 Years	105	50% (0.40-0.60)	61% (0.48-0.71)
5 Years	105	39% (0.29-0.49)	53% (0.39-0.65)
3 years survival after 1 year survival	65	65% (0.52-0.76)	79% (0.61-0.90)
3 years survival after 3 years survival	45	67% (0.50-0.79)	84% (0.55-0.95)
3 years survival after 5 years survival	31	63% (0.43-0.78)	82% (0.45-0.95)
Black			
1 Year	4	75% (0.13-0.96)	76% (0.12-0.96)
3 Years	4	5% (0.06-0.85)	57% (0.04-0.91)
5 Years	4	5% (0.06-0.85)	57% (0.04-0.91)
3 years survival after 1 year survival	3	67% (0.05-0.95)	73% (0.02-0.98)
3 years survival after 3 years survival	2	-	-
3 years survival after 5 years survival	2	-	-
Asian or Pacific islander			
1 Year	4	75% (0.13-0.96)	75% (0.13-0.96)
3 Years	4	75% (0.13-0.96)	75% (0.13-0.96)
5 Years	4	-	-
3 years survival after 1 year survival	3	-	-
3 years survival after 3 years survival	1	-	-
3 years survival after 5 years survival	0	-	-

Disclaimer/Publisher's Note: The statements, opinions and data contained in all publications are solely those of the individual author(s) and contributor(s) and not of MDPI and/or the editor(s). MDPI and/or the editor(s) disclaim responsibility for any injury to people or property resulting from any ideas, methods, instructions or products referred to in the content.
